# Vacuum-Assembled ZIF-67/SiO_2_–PEI Thin-Film Nanocomposite Membrane with Ultrahigh Permeance for Textile Wastewater Treatment

**DOI:** 10.3390/polym17131741

**Published:** 2025-06-22

**Authors:** Li Xiao, Jinyu Liu, Fan Zhang, Feng Qin, Yikai Wang, Zikang Qin, Yahui Yang, Zhongde Dai, Junfeng Zheng, Bo Tang

**Affiliations:** 1 CNOOC Key Laboratory of Liquefied Natural Gas and Low-Carbon Technology, Beijing 100028, China; 2 College of Ecology and Environment, Chengdu University of Technology, Chengdu 610059, China; 3 College of Carbon Neutrality Future Technology, Sichuan University, Chengdu 610065, China; 4 National Engineering Research Centre for Flue Gas Desulfurization, Chengdu 610065, China

**Keywords:** dye/salt separation, Thin-film nanocomposite membranes, ZIF-67, SiO_2_

## Abstract

High permeance combined with high salt/dye separation efficiency is a prerequisite for achieving zero-liquid-discharge treatment of saline textile wastewater by membrane technology. Thin-film nanocomposite (TFN) membranes incorporating porous nanoparticles offer a promising route to overcome the permeability–selectivity trade-off of conventional polymer membranes. In this study, a vacuum-assisted method was used to co-blend ZIF-67 and SiO_2_ nanoparticles, while branched polyethyleneimine (PEI) served as a cross-linking bridge, resulting in a high-performance TFN membrane for salt/dye separation. Acting as a molecular connector, PEI coordinated with ZIF-67 through metal–amine complexation and simultaneously formed hydrogen bonds with surface hydroxyl groups on SiO_2_, thereby linking ZIF-67 and SiO_2_. The resulting membrane exhibited good hydrophilicity and excellent dye separation performance (water flux = 359.8 L m^−2^ h^−1^ bar^−1^; Congo Red rejection = 99.2%) as well as outstanding selectivity in dye/salt mixtures (Congo Red/MgCl_2_ selectivity of 1094). The optimal ZIF@SiO_2_-PEI membrane maintained stable dye rejection over a wide range of trans-membrane pressures, initial concentrations, and pH values. These results reveal the huge potential of applying the ZIF@SiO_2_-PEI TFN membranes for resource recovery in sustainable textile wastewater systems.

## 1. Introduction

Textile printing and dyeing effluents have become a major source of water pollution in recent years. To improve dye purity and fabric fixation, large quantities of inorganic salts are routinely added during dyeing processes [1,2]. Improper discharge of these saline, dye-laden wastewaters inflict irreversible damage on aquatic ecosystems and wastes valuable resources [1,3]. Consequently, efficiently separating salts from dye/salt mixtures is essential for dye recovery and reuse, and methods capable of fractionating dyes and inorganic salts are drawing increasing attention [4,5].

Membrane separation is a practical, easily controlled method for water purification owing to its straightforward construction and process operation [6]. Although eliminating all pollutants simultaneously is relatively straightforward, the current research challenge and focal point is how to separate different pollutants in wastewater so that valuable components can be recovered and true zero discharge can be achieved [7]. Conventional processes such as reverse osmosis (RO) and nanofiltration (NF) can remove both salts and dyes, whereas ultrafiltration (UF) membranes show insufficient dye rejection. In recent years, loose nanofiltration membranes have been introduced to separate salts from dyes. High contaminant rejection and outstanding permeability are two crucial factors in membrane separation; however, due to the intrinsic limitations of polymeric materials, fabricating membranes that combine high permeability with high rejection remains a challenge [8].

Metal–organic frameworks (MOFs), a class of highly ordered crystalline porous materials, possess exceptionally high porosity and tunable pore apertures, granting them tremendous potential in separation science [9]. In recent years, as an understanding of MOF chemistry has deepened and hydrolytic stability has improved, MOF-based membranes have been extensively employed for liquid-phase solute separations. Within this family, zeolitic imidazolate frameworks (ZIFs) offer exceptionally high surface areas, diverse topologies, and tunable pore apertures [10]. In recent years, there have been many studies on dye removal by MOF-based membranes [2]. However, membranes fabricated solely from MOFs lack adequate mechanical strength and exhibit limited long-term stability. Accordingly, current research focuses on two strategies: embedding MOFs into polymer matrices to create mixed-matrix membranes (MMMs) and depositing MOF-based thin-film nanocomposite (TFN) layers onto porous supports.

To couple high permeance with sharp selectivity, Yang et al. fabricated biocatalytic MMMs in which polydopamine (PDA) served as an interfacial “bridge” between MOF domains and immobilised laccase. The resulting PVDF-based MMMs, featuring a continuous MOF network, alleviated the classical permeability–selectivity trade-off and achieved efficient dye/salt fractionation [11]. Wang et al. subsequently developed a thermally induced phase-separation hot-pressing (TIPS-HoP) strategy for roll-to-roll production of ten distinct MOF membranes; interweaving ultra-high-molecular-weight polyethylene with MOF particles endowed the films with high mechanical strength and 99% rejection of organic dyes at a flux of L m^−2^ h^−1^ bar^−1^ under cross-flow operation [12]. Wu and co-workers employed non-solvent-induced phase separation to create a porous PVDF/poly(vinylpyrrolidone) support, followed by sequential deposition of chitosan, poly(vinyl alcohol), and ZIF-8, yielding continuous symbiotic MMMs that effectively removed organic dyes and heavy-metal ions [13]. While these advances have markedly improved MOF-based membrane performance, further enhancements in water flux, dye/salt selectivity, chemical stability, and antifouling resistance remain pressing research priorities.

Silica (SiO_2_) nanoparticles are inexpensive, easy to process, and intrinsically hydrophilic; consequently, their incorporation into membranes can markedly improve surface wettability, thereby increasing water flux and enhancing antifouling performance. Building on these advantages, we devised a facile vacuum-assisted assembly that simultaneously embeds ZIF-67 and SiO_2_ into a porous nylon substrate, yielding a series of TFN membranes. Branched polyethyleneimine (PEI) serves as a molecular cross-linker, coordinating with ZIF-67 and hydrogen bonding to SiO_2_ to stabilise an integrated hybrid network and enhance chemical robustness. The resulting ZIF@SiO_2_ TFNs were systematically characterised for surface composition, hydrophilicity, charge density, morphology and filler topology. Their performance was subsequently evaluated through pure-water permeance measurements and rejection tests using representative dyes and inorganic salts. These investigations clarify the bridging role of PEI, reveal the synergy between MOF porosity and silica hydrophilicity, and establish explicit structure–property–performance relationships for this new membrane platform.

## 2. Materials and Methods

### 2.1. Materials

Methyl Blue (Mw ≈ 799.8 g mol^−1^, BS grade), 2-methylimidazole (≥99%, LR), Congo Red (Mw ≈ 696.7 g mol^−1^, AR), Direct Red 80 (Mw ≈ 1373 g mol^−1^, AR), and branched polyethyleneimine (PEI, Mw ≈ 10 kDa, 99%) were purchased from Chengdu Kelong Chemical Co., Ltd., Chendu, China. Sodium hydroxide pellets (≥98%, AR), magnesium chloride hexahydrate (MgCl_2_·6H_2_O, 99%, AR), and hydrophilic nylon microfiltration membranes were supplied by Shanghai Titan Scientific Co., Ltd., Shanghai, China. Direct Blue 15 (Mw ≈ 882.8 g mol^−1^, BS), cobalt(II) nitrate hexahydrate (Co(NO_3_)_2_·6H_2_O, ≥99%, AR), and hydrophilic fumed silica (BET surface area ~380 m^2^ g^−1^; primary particle size 7–40 nm) were obtained from Shanghai Aladdin Biochemical Technology Co., Ltd., Shanghai, China. De-ionised water (18.2 MΩ cm) was used throughout. All reagents were employed as received without further purification.

### 2.2. Synthesis of ZIF-67

ZIF-67 nanoparticles were prepared by facile room-temperature precipitation [14]. Cobalt(II) nitrate hexahydrate (0.145 g, 0.50 mmol) and 2-methylimidazole (0.481 g, 5.85 mmol) were each dissolved in de-ionised water (100 mL) under magnetic stirring. The two solutions were then combined and stirred at 550 rpm for 20 min at ambient temperature, yielding a homogeneous blue suspension. The mixture was allowed to stand undisturbed for a further 60 min to complete crystallisation, affording a ZIF-67 dispersion with a solids concentration of 1.35 mg mL^−1^. The dispersion was stored at 4 °C and used without further purification.

### 2.3. Fabrication of TFN Membranes

#### 2.3.1. Fabrication the ZIF67 Membrane

**Figure 1:**
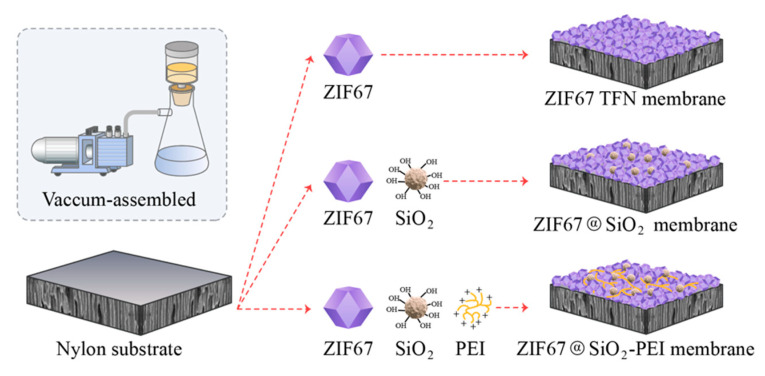
Schematic representation of constructing TFN membranes.

As illustrated in Figure 1, ZIF67 TFN membranes with graded crystal loadings were produced by vacuum-filtering pre-synthesised ZIF-67 suspensions of different volumes onto 3 cm diameter porous nylon supports that had been pre-soaked in de-ionised water for 24 h to enhance surface wettability and promote strong interfacial adhesion. The water flux and dye/salt rejection of each membrane were screened (Section 3.2), and the formulation containing 0.075 g ZIF-67 yielded the best overall performance; this composition was therefore adopted for all subsequent work.

#### 2.3.2. Fabrication of ZIF@SiO_2_ TFN Membranes

Hydrophilic SiO_2_ nanoparticles (0.200 g) were dispersed in de-ionised water (100 mL) and stirred at 450 rpm for 20 h to obtain a stable suspension. Defined aliquots of this suspension were added to the optimised ZIF-67 dispersion (Section 2.3.1), followed by 1 min of ultrasonication to ensure uniform mixing. The resulting slurry was deposited onto nylon supports by vacuum filtration, affording TFN membranes with different silica loadings, hereinafter denoted ZIF@SiO_2_ TFN membrane. The equation was used to compute the incorporation of SiO_2_:

(1) $$w=\left(\frac{x}{x+y}\right)\times 100\%$$

Of these, “*x*” and “*y*” represent mass (g) of SiO_2_ and ZIF-67, respectively. Performance screening identified the membrane containing 2% SiO_2_ as optimal (see Section 3.2 for discussion).

#### 2.3.3. Preparation of ZIF@SiO_2_-PEI Membranes

Branched PEI (0.200 g) was dissolved in de-ionised water (100 mL) to form a stock solution. Aliquots of this solution were introduced into the ZIF@SiO_2_ (2%) dispersion, followed by 1 min ultrasonication. The mixture was then vacuum-filtered onto nylon supports to generate TFN membranes with varying PEI contents, designated ZIF@SiO_2_-PEI membrane. The calculation of PEI loading was performed using specific Equation (2):

(2) $$w=\left(\frac{x}{x+y+z}\right)\times 100\%$$

Here, *z* represents the quality level of the PEI (g), *x* represents the SiO_2_′ quality (g), and *y* represents the quality level of the ZIF-67 (g).

### 2.4. Characterization Methods

The porosity and specific surface area of ZIF-67 were evaluated via N_2_ adsorption/desorption isotherms (ASAP 2460, Micromeritics, Norcross, GA, USA) using the Brunauer–Emmett–Teller (BET) method. Crystalline structure was identified by X-ray diffraction (XRD, SmartLab SE, Rigaku, Japan; Cu Kα radiation, 2θ = 5–60°, step size = 0.02°), while functional groups were probed using Fourier-transform infrared spectroscopy (FT-IR, Frontier, PerkinElmer, Waltham, MA, USA; 4000–600 cm^−1^). Elemental composition and chemical states were analysed by X-ray photoelectron spectroscopy (XPS, K-Alpha, Thermo Scientific, Waltham, MA, USA). Surface charge was determined with a zeta-potential analyser (Litesizer 500, Anton Paar, Graz, Austria). Membrane morphology was inspected by field-emission scanning electron microscopy (FESEM, Nova NanoSEM 450, FEI, Washington County, OR, USA) after sputter-coating the samples with gold for 60 s. Static water-contact angles were measured on a drop-shape analyser (DSA 25E, KRÜSS, Hamburg, Germany) to assess surface wettability.

### 2.5. Assessing the Efficacy of Membrane Separation

Filtration experiments were carried out on a cross-flow system (effective membrane area = 7.07 cm^2^). Prior to each run, the membrane was compacted with de-ionised water at 3 bar for 10 min to ensure flux stabilisation, after which the feed reservoir was charged with the desired dye solution and the trans-membrane pressure reset to the target value. Water permeance (*P*) and dye rejection (*R*) were calculated according to Equations (3) and (4), respectively [15].

(3) $$P=\frac{V}{S\times t\times \Delta P}$$

(4) $$R=\left(1-\frac{C_{p}}{C_{f}}\right)\times 100\%$$

In this context, *V* symbolises the permeation volume (L), *S* represents the region covered by the membrane cell (m^2^), *t* represents the operational duration (h), and *ΔP* represents the force exerted across the membrane (bar). The amount of dye present at the entry and exit points is *C_f_* and *C_p_* in that order.

Salt concentration was obtained using a conductivity tester (DDSJ-308A, Shanghai Yidian Scientific Instruments, Shanghai, China). Next, the selectivity (S) of the dye and salt can be calculated with Equation [16]:

(5) $$S=\frac{R_{dye}}{R_{salt}}\times 100$$

where *R_dye_* and *R_dye/salt_* are dye and salt exclusions, respectively. To evaluate the enduring stability of the membrane specimens, we underwent filtration with 50 mg·L^−1^ of CR solution, maintaining a feed pressure of 1 bar.

## 3. Results and Discussion

### 3.1. Membrane Characterisation

**Figure 2:**
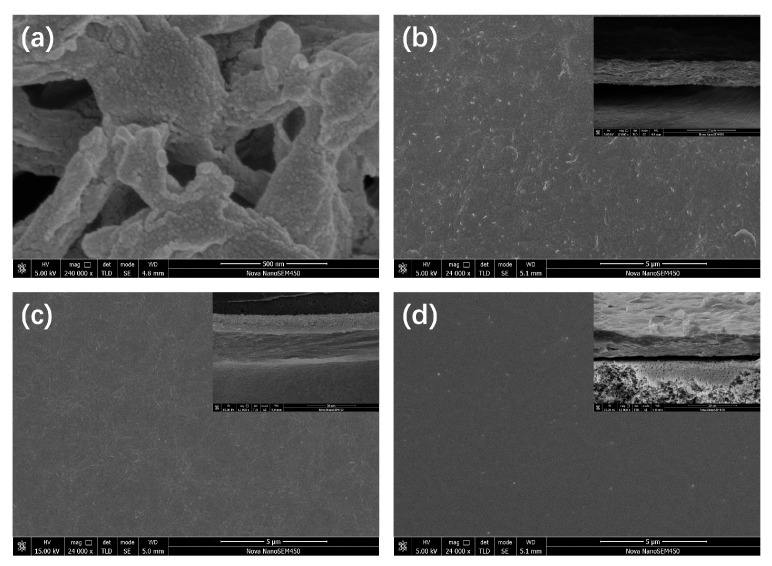
FESEM images of (**a**) porous nylon substrate; (**b**) ZIF-67 TFN membrane (surface and cross-section); (**c**) ZIF@SiO_2_ TFN membrane (surface and cross-section); and (**d**) ZIF@SiO_2_-PEI TFN membrane (surface and cross-section).

Representative FESEM micrographs are compiled in Figure 2, Figures S1 and S2. The pristine nylon support displays an open, interconnected pore structure (Figure 2a). After vacuum filtration of the ZIF-67 dispersion, these pores are completely occluded and a continuous polycrystalline ZIF-67 layer is formed (Figure 2b, surface and cross-section), confirming successful deposition. Particle-level details of the hydrophilic silica are provided in Figure S1a, where the fumed SiO_2_ appears as agglomerated primary particles with diameters of ~40 nm. Introducing SiO_2_ produces discernible silica domains on the membrane surface (Figure 2c), increasing microscale roughness; the corresponding cross-section (Figure S1b,c) reveals a more loosely packed ZIF-67 layer, a morphology that is expected to enhance hydrophilicity. Subsequent incorporation of PEI (ZIF@SiO_2_-PEI) further modifies the topography, generating small surface voids while retaining the relaxed layered structure (Figure 2d).

**Figure 3:**
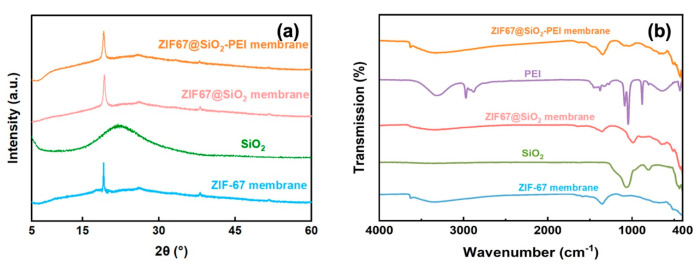
(**a**) XRD pattern and (**b**) FTIR of SiO_2_, ZIF-67, ZIF@SIO_2_, and ZIF@SIO_2_-PEI.

Beyond morphological inspection, the composite structure was verified by XRD and FT-IR. As shown in Figure 3a, ZIF-67 membranes exhibit a sharp reflection at 2θ ≈ 18.5°, corresponding to the (222) plane of the ZIF-67 reference pattern [17]. This peak remains unchanged in the ZIF@SiO_2_ and ZIF@SiO_2_-PEI profiles, confirming that neither silica addition nor PEI cross-linking perturbs the ZIF-67 lattice. A broad halo centred at 2θ ≈ 23° is evident in the silica-containing samples and is attributable to amorphous SiO_2_. FT-IR spectra provide complementary chemical evidence [18,19]. The band at 965 cm^−1^ originates from Si–OH vibrations, while the intense feature at 1098 cm^−1^ corresponds to the antisymmetric stretch of Si–O–Si [19]. Incorporation of PEI is signalled by a new C–N stretching band at 1278 cm^−1^ and by amine vibrations at 1647 cm^−1^ (secondary) and 1568 cm^−1^ (primary); methylene stretches of the PEI backbone appear at 2970 and 2879 cm^−1^ [20]. Collectively, the XRD and FT-IR data confirm the successful integration of SiO_2_ and PEI into the ZIF-67 framework without compromising its crystallinity, thereby establishing the intended hybrid architecture of the TFN membranes.

XPS provided definitive evidence for the stepwise construction of the hybrid membranes. The survey spectrum of the parent ZIF-67 TFN shows characteristic Co 2p, N 1s, O 1s, and C 1s peaks at ~784, 401, 533, and 287 eV, respectively, while high-resolution Co 2p reveals Co^2+^ doublets at 783.2 eV (2p_3_/_2_) and 799.1 eV (2p_1_/_2_) accompanied by shake-up satellites at 788.6 and 805.0 eV, in agreement with the ZIF-67 lattice [21]. Deconvolution of the O 1s envelope yields components at 532.2 and 533.5 eV, assigned to O–C and O–Co bonds. After silica incorporation, a Si 2p doublet appears at 102.3 and 103.9 eV in the ZIF@SiO_2_ membrane, corresponding to Si–C and Si–O–Si environments [22]; the negligible shift of the Si–O component in O 1s (ΔB.E. ≈ 0.9 eV) and the disappearance of the C–O contribution indicate that SiO_2_ acts as an inert filler without perturbing the ZIF-67 framework. Subsequent PEI grafting significantly intensifies the N 1s signal and introduces a new peak at ~399.3 eV, attributable to amine/pyridinic nitrogen from PEI, while the Co 2p and Si 2p features remain intact, confirming that polyamine chains are successfully anchored through coordinative and hydrogen-bond interactions. Together with SEM, XRD, and FT-IR results, these XPS findings substantiate the successful integration of SiO_2_ and PEI into the ZIF-67 matrix and the preservation of the intended crystalline–amorphous hybrid architecture.

Hydrophilicity strongly influences membrane water flux and fouling resistance; it is commonly assessed by the water-contact angle (WCA), which decreases as surface affinity for water increases. Figure S3a shows that all TFN formulations exhibit WCAs below 90°, confirming intrinsically hydrophilic surfaces. The pristine ZIF-67 membrane displays a WCA of 40.7°. Incorporation of hydrophilic silica nanoparticles further lowers this value: At 3 wt % SiO_2_, the WCA reaches 35.1°, in agreement with earlier reports that inorganic nanofillers enhance surface wettability [18]. By contrast, introducing branched polyethyleneimine raises the WCA slightly; the membrane containing 1 wt % PEI records 48.9°, and the angle increases marginally with additional PEI. The modest rise is attributed to the denser cross-linked network formed by excess polyamine, which reduces surface porosity and limits water penetration. Overall, silica doping improves hydrophilicity, whereas high PEI loadings exert the opposite effect by tightening the selective layer.

### 3.2. Optimisation of Membrane Separation Performance

#### 3.2.1. Optimising the ZIF67 Membranes

**Figure 4:**
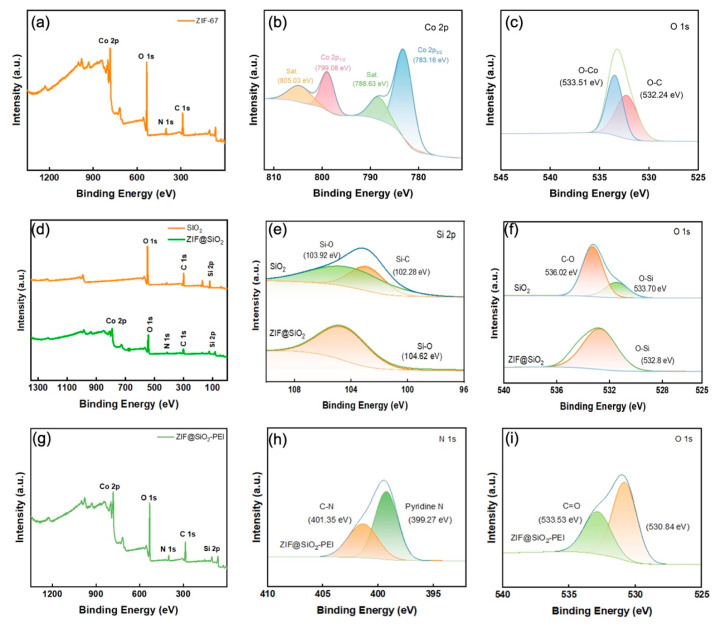
(**a**) XPS spectra of ZIF-67 membrane; (**b**,**c**) high-resolution curve-fitted spectra of Co 2p and O 1s; (**d**) XPS survey spectra of SiO2 and ZIF@SIO_2_; (**e**,**f**) high-resolution curve-fitted spectra of Si 2p, O 1s; (**g**) XPS survey spectra of ZIF@SIO_2_-PEI; and (**h**,**i**) high-resolution curve-fitted spectra of N 1s, O 1s.

Figure 4a presents the dependence of pure-water permeance on the mass of ZIF-67 deposited. Increasing the loading from 0.05 to 0.125 g systematically reduces permeance because the resultant thicker selective layer lengthens the diffusion path and elevates hydraulic resistance [23]. At any given loading, permeance declines with increasing dye molecular weight, confirming that size-sieving governs solute transport. The rejection trend is the opposite (Figure 4b): Higher ZIF-67 contents tighten the membrane structure, and at 0.125 g all four dyes—Methyl Blue, Direct Blue 15, Direct Red 80, and Congo Red—are rejected by >98%. Balancing flux and selectivity, a loading of 0.075 g yields the optimum trade-off, affording 99.1% Congo Red rejection while sustaining a water permeance of 175 L m^−2^ h^−1^ bar^−1^.

#### 3.2.2. Optimising the ZIF67@SiO_2_ Membranes

The influence of silica incorporation on membrane performance was examined using dye feeds of decreasing molecular weight—Direct Red 80, Direct Blue 15, Methyl Blue, and Congo Red—and the results are summarised in Figure 4c,d. As the SiO_2_/ZIF-67 mass ratio increased, pure-water permeance rose continuously, a trend attributed to the marked reduction in contact angle upon doping with hydrophilic silica, which lowers the energetic barrier for water uptake and transport. Concomitantly, dye rejection declined slightly; nonetheless, all ZIF@SiO_2_ membranes maintained rejections above 92%. When the silica fraction exceeded 1%, the rejection drop became pronounced, likely because excess SiO_2_ agglomerated to create micro-voids that compromised the structural integrity of the selective layer [24]. An optimal balance was achieved at 2 wt % SiO_2_: Permeances of 301.0, 314.1, 320.1 and 371.6 L m^−2^ h^−1^ bar^−1^ were recorded for Direct Red 80, Direct Blue 15, Methyl Blue, and Congo Red, respectively, alongside rejections of 97.4, 93.3, 97.0, and 97.8%. These data confirm that moderate silica loading enhances hydrophilicity and flux without sacrificing dye/salt selectivity.

#### 3.2.3. Optimising the ZIF67@SiO_2_-PEI Membranes

**Figure 5:**
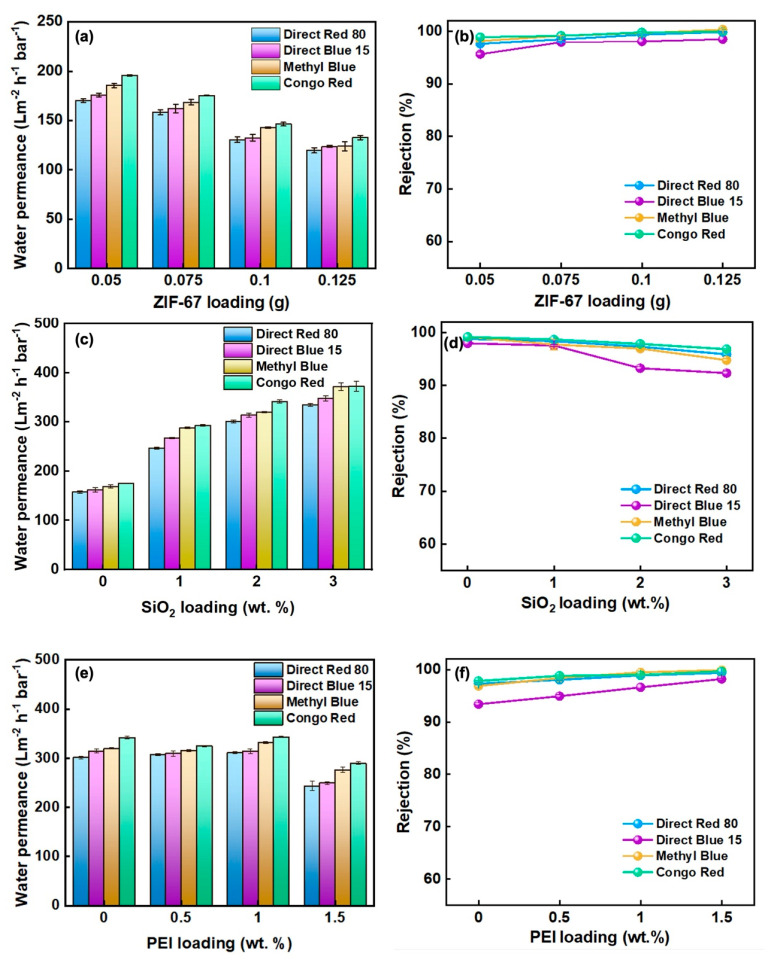
(**a**,**b**) Water flux and dye rejection rate of ZIF-67 membranes, (**c**,**d**) ZIF@SIO_2_ membranes, and (**e**,**f**) ZIF@SiO_2_-PEI membranes.

The effect of PEI cross-linking was assessed by introducing varying amounts of PEI into the ZIF@SiO_2_ (2%) formulation and evaluating flux and rejection towards dyes of different molecular weights (Figure 5e,f). Increasing the PEI content from 0 to 1 wt % produced only a minor change in water permeance, but a further increase to 1.5 wt % caused a sharp decline; e.g., the flux for DR 80 fell to 244 L m^−2^ h^−1^ bar^−1^ because excess polyamine densified the selective layer and narrowed water-transport pathways, thereby elevating hydraulic resistance [25]. In contrast, dye rejection improved progressively with PEI loading (Figure 5f). The enhancement is attributed to two cooperative effects: (i) PEI coordinates to Co^2+^ centres in ZIF-67 and forms hydrogen bonds with silanol groups on SiO_2_, tightening the interlayer spacing and sealing defects, and (ii) the protonatable amine groups introduce additional positive charge, strengthening electrostatic exclusion of the anionic dyes. The optimum balance was achieved at 1 wt % PEI, where the membrane delivered a permeance of 362 L m^−2^ h^−1^ bar^−1^ together with 99.4% rejection of Methyl Blue, evidencing the synergistic contribution of size-sieving and charge repulsion.

#### 3.2.4. Effect of Salt Content in Dye/Salt Separation Process

**Figure 6:**
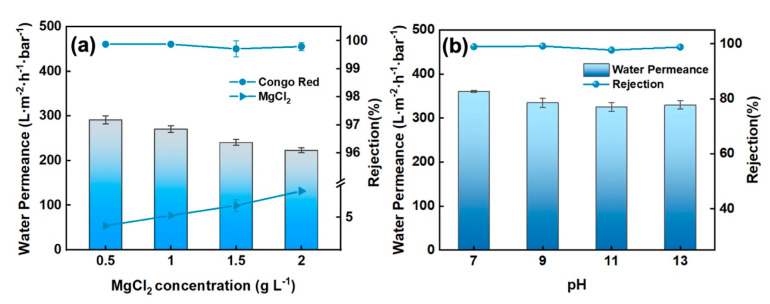
Separation performance of ZIF@SIO_2_-PEI membrane with (**a**) MgCl_2_ concentration and (**b**) pH.

The ZIF@SiO_2_-PEI membrane was challenged with mixed feeds containing 50 mg L^−1^ Congo Red and 0–2000 ppm MgCl_2_ (Figure 6a). Permeance declined monotonically as MgCl_2_ concentration increased, consistent with the heightened osmotic pressure and concentration-polarisation commonly observed for loose layers at elevated ionic strength [26,27,28]. Congo Red rejection, however, remained essentially constant at >99% throughout, demonstrating that size-sieving combined with electrostatic exclusion is largely insensitive to bulk salinity [29]. Magnesium rejection increased slightly with salt concentration, yet remained below 10% even at 2000 ppm, because the higher surface concentration of Mg^2+^ accentuates electrostatic repulsion inside the membrane pores. The resulting separation factor of CR/MgCl_2_ reached 1094, underscoring the membrane’s excellent selectivity for dye over divalent salt in saline feeds [30].

#### 3.2.5. Effect of pH on Separation Performance

Solution pH modulates both membrane surface charge and dye ionisation; therefore, separation performance was assessed over pH 7–14 (Figure 6b). Water permeance decreased progressively with increasing pH, a trend attributed to the greater hydrophobic character of Congo Red under alkaline conditions, which fosters dye adsorption and partially blocks water-transport pathways [31]. Concurrently, dye rejection deteriorated as pH rose. This behaviour is consistent with a Donnan-controlled mechanism: Deprotonation of the feed at high pH diminishes the positive charge density of the ZIF@SiO_2_-PEI surface, thereby weakening electrostatic repulsion between the anionic dye molecules and the membrane and lowering overall rejection [32]. The data collectively indicate that the membrane performs optimally under neutral to mildly alkaline conditions, where electrostatic exclusion and size-sieving act synergistically [33].

#### 3.2.6. Antifouling and Stability Performance of the TFN Membrane

**Figure 7:**
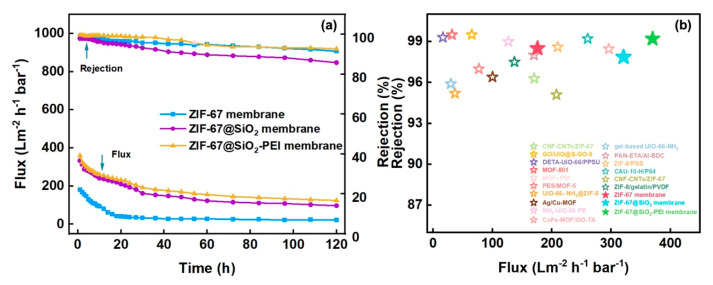
(**a**) Long-term stability of ZIF-67, ZIF@SIO_2_-PEI, and ZIF@SIO_2_-PEI TFN membranes within a 50 mg L^−1^ Congo Red solution. (**b**) Analysing the performance of TFN membranes against data.

Operational stability was assessed by filtering a 50 mg L^−1^ Congo Red solution through the ZIF-67, ZIF@SiO_2_, and ZIF@SiO_2_-PEI membranes at 1 bar for 120 h (Figure 7a). All three membranes exhibited a rapid flux decline during the first 16 h as dye molecules adsorbed on and within the selective layer, after which the flux stabilised. After 120 h, the fluxes of the ZIF-67, ZIF@SiO_2_, and ZIF@SiO_2_-PEI membranes levelled at 22, 97, and 125 L m^−2^ h^−1^ bar^−1^, respectively, while the corresponding Congo Red rejections were 91.5, 85.8, and 92.7%. The rejection similarity between ZIF-67 and ZIF@SiO_2_-PEI—and the noticeably lower rejection of ZIF@SiO_2_—reflects interfacial compatibility: Without the PEI bridge, a limited affinity between ZIF-67 and SiO_2_ introduces microscopic defects that compromise selectivity. In contrast, the flux profiles show the opposite trend: ZIF@SiO_2_ and ZIF@SiO_2_-PEI deliver comparable high permeance, whereas the pristine ZIF-67 membrane exhibits roughly half that value because it lacks the hydrophilic silica pathways that facilitate water transport. Collectively, these results confirm a synergistic effect: The ZIF@SiO^2^-PEI membrane combines the high rejection characteristic of the ZIF-67 layer with the high flux imparted by silica, while PEI cross-linking eliminates the incompatibility-induced defects that otherwise diminish performance [34].

To contextualise these results, Table 1 compiles the flux, dye rejection and salt rejection reported for state-of-the-art dye/salt separation membranes. As plotted in Figure 7b, the ZIF@SiO_2_-PEI membrane delivers one of the highest fluxes among comparable systems while maintaining > 92% dye rejection and <10% MgCl_2_ rejection. This combination of high throughput, sharp selectivity and long-term stability underscores the membrane’s promise for practical zero-liquid-discharge treatment of saline textile wastewater [35].

## 4. Conclusions

In this study, we demonstrate a TFN membrane in which ZIF-67 nanosheets, hydrophilic silica nanoparticles, and a PEI molecular bridge act synergistically to break the traditional permeability–selectivity trade-off. The optimised ZIF@SiO_2_-PEI membrane delivers a water permeance of 370 L m^−2^ h^−1^ bar^−1^ while rejecting 99% of Congo Red and <10% of magnesium chloride, a CR/MgCl_2_ selectivity of 1094. The membrane maintains >95% of its initial flux after 24 h continuous filtration of dye–salt feeds, highlighting its operational robustness. Silica introduces rapid hydration pathways, and PEI coordinatively links ZIF-67 to SiO_2_, sealing inter-crystalline voids without compromising porosity. These findings establish a modular route to high-flux, dye-selective membranes suited to zero-liquid-discharge treatment of saline textile effluents. Rational tuning of MOF chemistry and interlayer spacing should now enable discrimination among smaller organics and multivalent ions, extending the reach of MOF-based membranes across industrial water-reuse sectors.

## Figures and Tables

**Table 1:** Assessing the effectiveness of separating dyes in different MOF-based membranes.

Membranes	Dye	Weight of a Molecule	Permeance (L m^−2^ h^−1^ bar^−1^)	R (%)	Ref
GO/UiO@S-GO-8	Methylene Blue	320	65.1	99.5	[36]
DETA-UiO-66/PPSU	Reactive Orange 16	617	16.5	99.3	[2]
MOF-801	Congo Red	696	31.7	99.5	[37]
Fio-TFN	Rhodamine B	479	255.9	82.0	[38]
PES/MOF-5	Methylene Blue	320	77.1	97.0	[39]
UiO-66-NH_2_@ZIF-8	Direct Red 80	1373	36.7	95.2	[40]
Ag/Cu-MOF	Reactive Black 5	992	100.0	96.4	[41]
NH_2_-UiO-66 PE	Congo Red	696	126.9	99.0	[12]
CoFe-MOF/GO-TA	Rhodamine B	479	296.2	98.5	[42]
gel-based UiO-66-NH_2_	sunset yellow	452	29.7	95.9	[43]
PAN-ETA/Al-BDC	Congo Red	696	170.0	98.0	[44]
ZIF-8/PSS	Methyl Blue	799	210.0	98.6	[45]
CAU-10-H/P84	Methyl Blue	799	260.4	99.2	[46]
ZIF-67	Congo Red	696	175.8	98.5	This work
ZIF@SIO_2_-PEI	Methyl Blue	799	321.0	97.9	
ZIF@SIO_2_-PEI	Congo Red	696	369.8	99.2	

## Data Availability

The original contributions presented in this study are included in this article/Supplementary Materials. Further inquiries can be directed to the corresponding author.

## Supplementary Materials

The following supporting information can be downloaded at: https://www.mdpi.com/article/10.3390/polym17131741/s1, Figure S1. (a) FESEM images of SiO_2_ nanoparticles; cross-sectional FESEM images of (b) ZIF-67, (c) ZIF@SiO_2_, and (d) ZIF@SiO_2_-PEI TFN membrane. Figure S2. Higher-magnification FESEM images (200,000×) of (a) porous nylon substrate; (b) ZIF-67; (c) ZIF@SiO_2_; and (d) ZIF@SiO_2_-PEI TFN membrane. Figure S3. (a) Water-contact angles. (b) Zeta potential of ZIF-67, SiO_2_, ZIF@SIO_2_, and ZIF@SIO_2_-PEI membrane.
