# Supplementary material for: Vacuum-Assembled ZIF-67/SiO2–PEI Thin-Film Nanocomposite Membrane with Ultrahigh Permeance for Textile Wastewater Treatment
Source: Polymers (Basel). 2025 Jun 22;17(13):1741. doi: 10.3390/polym17131741 (PMC12251891; doi:10.3390/polym17131741)
Supplement: Supplementary file 1 [file polymers-17-01741-s001.zip › polymers-3704572-supplementary.pdf]

Supporting Information

Vacuum-Assembled ZIF-67/SiO_2–PEI Thin-Film Nanocomposite Membrane with Ultrahigh Permeance for Textile Wastewater Treatment

Li Xiao[1], Jinyu Liu [2, 3], Fan Zhang[1], Feng Qin[1], Yikai Wang[3,4], Zikang Qin [3,4], Yahui Yang [2], Zhongde Dai [3,4], Junfeng Zheng [1, 3,4]*, Bo Tang [2,5]*

[1]CNOOC Key Laboratory of Liquefied Natural Gas and Low-Carbon Technology, Beijing 100028, China

[2] College of Ecology and Environment, Chengdu University of Technology, Chengdu 610059, China.

[3] College of Carbon Neutrality Future Technology, Sichuan University, Chengdu 610065, China.

[4] National Engineering Research Centre for Flue Gas Desulfurization, Chengdu 610065, China.

*Corresponding Author:

Junfeng Zheng, junfeng.zheng@scu.edu.cn

Bo Tang, tangbo18@cdut.edu.cn

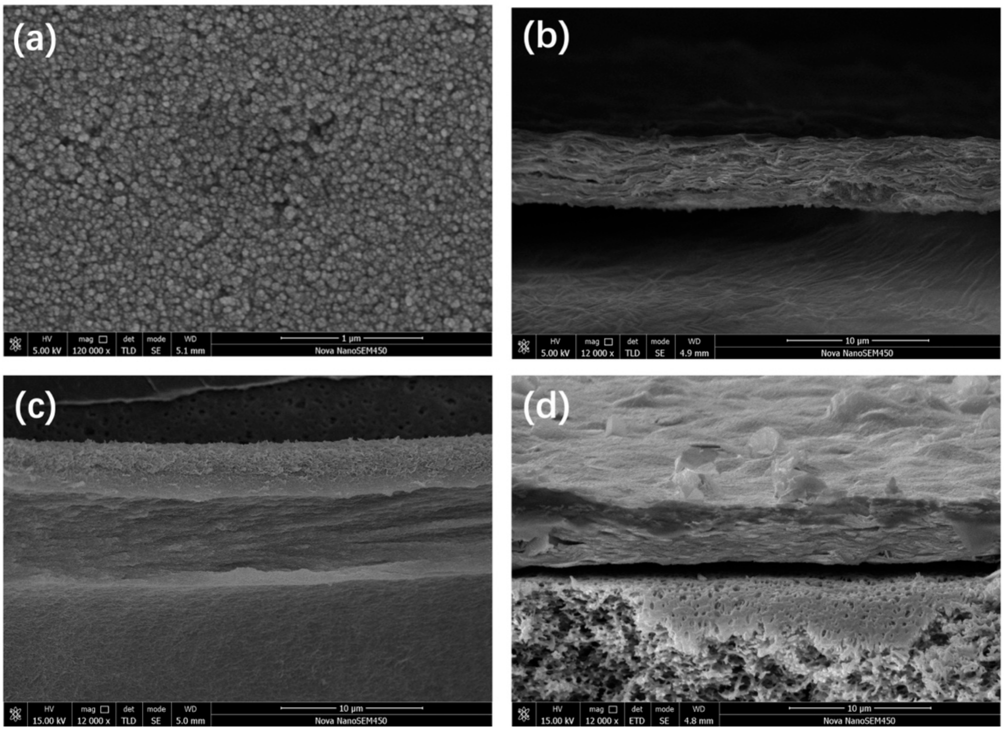

Figure S1. (a) FESEM images of SiO_2 nanoparticles; Cross-sectional FESEM images of (b) ZIF-67, (c) ZIF@SiO_2 and (d) ZIF@SiO_2-PEI TFN membrane.

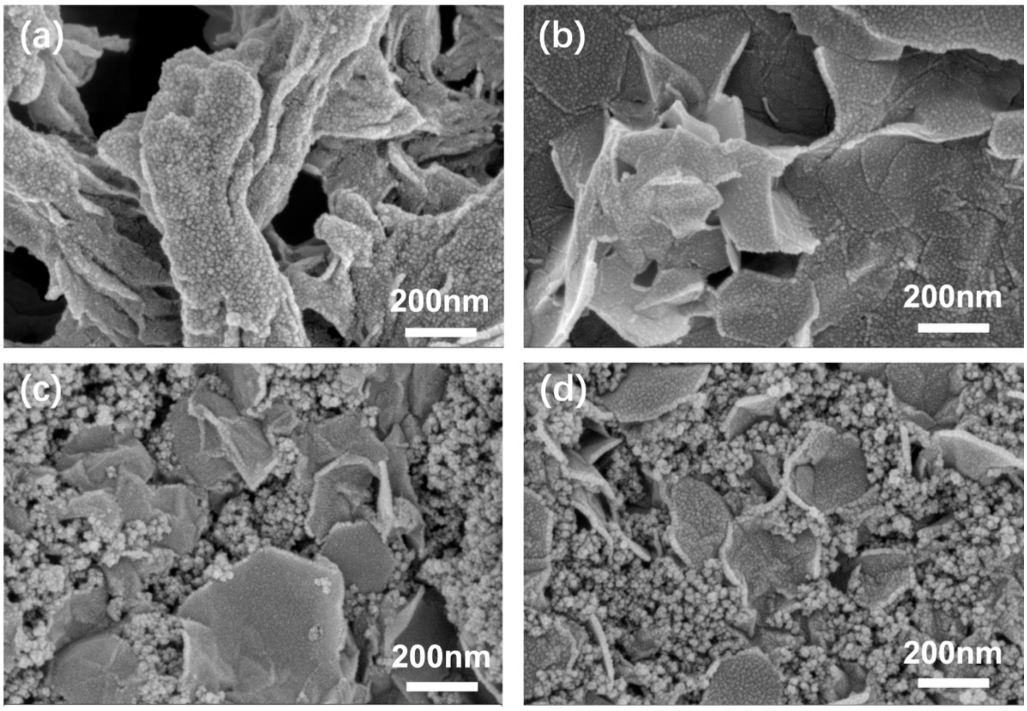

Figure S2. Higher-magnification FESEM images (200 000×) of (a) porous nylon substrate; (b) ZIF-67, (c) ZIF@SiO_2 and (d) ZIF@SiO_2-PEI TFN membrane.

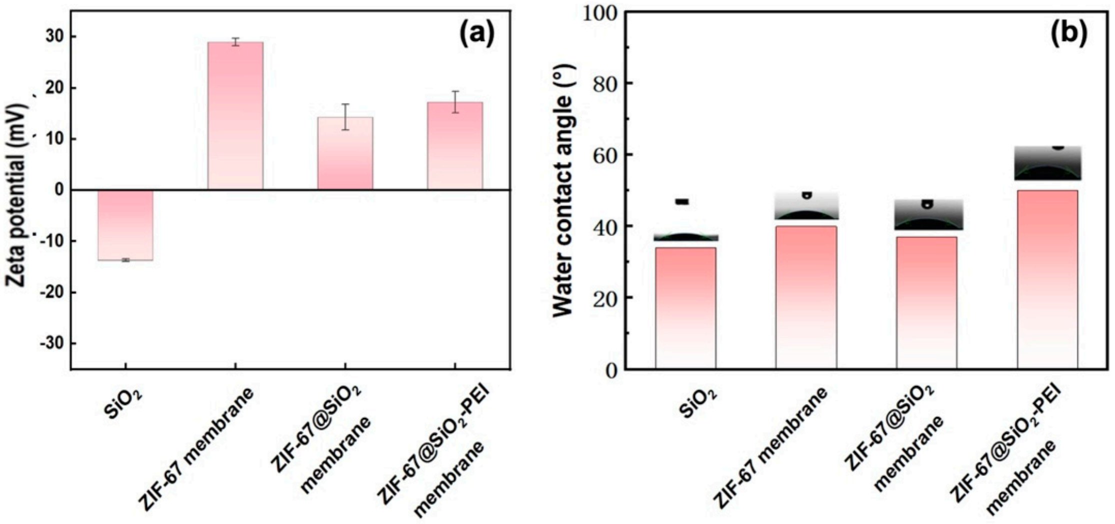

Figure S3. (a) Water contact angles (b) Zeta potential of ZIF-67, SiO_2, ZIF@SIO_2 and ZIF@SIO_2-PEI membrane.
